# Report of the Proceedings of the Medical Societies of New York

**Published:** 1858-02

**Authors:** 

**Affiliations:** President


From the New York Journal of Medicine.

*Report of the Proceedings of the Medical Societies of New York.*

NEW YORK ACADEMY OF MEDICINE.

REGULAR MEETING, DEC. 2, 1857, DR. MOTT, *President.*

PUERPERAL FEVER.

DR. A. CLARKE said:—That as much time had elapsed since he was first honored with the attention of the Academy on this subject, he would take the liberty of recapitulating the positions he had taken in regard to the nature of puerperal fever. He had expressed his concurrence in the views of those who believe the disease to be contagious, and had spent a little time in considering the different doctrines relating to the means by which the contagion is conveyed from one person to another. He had stated that in his belief the disease is composed of two elements, a fever and an inflamation. In this respect, it resembles the epidemic dysentery, the epidemic erysipelas, or small pox. He had already stated that in the epidemic erysipelas which prevailed in New England, and in the western States, from seven to ten years ago, these two elements were as clearly distinct, in the time of their developement, as they are in small pox. A febrile movement, lasting a variable time, attended by swelling of tonsils, and of the superficial and deepseated glands of the neck, preceded the grave inflammatory lesion; and when this latter made its appearance, one, two, or three days after the fever commenced, it was an erysipelas of the head and face, of the lower extremities, the body, side, or arm; or it was pleurisy without any external inflammation, or a peritonitis, or indeed, almost any internal inflammation. In lying-in women, and those in the puerperal state, it assumed the form of puerperal fever. In these various modes of developement, it seemed as if the same poison was demonstrating its morbific power bylighting up a fever first, and kindling an inflammation afterwards, the double effect of one agent. He had expressed his belief that the fever of the puerperal disease was just as certainly followed by inflammation, as the fever of small pox is followed by an eruption, complete or incomplete; and as the fever of small pox never endangers life till the eruption, or some modification of it occurs, so he believed the puerperal disease is never fatal, but by the aid of its inflamatory element.—These comparisons, however, illustrate nothing more than the compound nature of this fever, the succession in the two leading elements follows a very different law; for while small pox fever requires two days to reach its eruptive stage, and epidemic erysipelas one to three days to reach its inflammation, the puerperal fever, like epidemic dysentery is followed promptly by its local lesion, commonly in a few hours, often in one. He believed in the communicable character of the disease, and this independent of other reasons would almost compel him to believe in its febrile nature. He believed in the inflammatory lesions, because their constancy is testified to by every accredited observer, except in a few rare instances.

These rare instances were the cases, which, in the belief of Simpson, Gooch, and others, were without lesion of any kind—a simple fever, the poison of which overwhelmed the vital powers. It was his object, on a former occasion, to show that these cases were no exceptions to the general rule, but that they were really marked by inflammation, like the others, but that the inflammation was one that had escaped detection, that it was an endometritis, and that the inflammation affecting the inner surface of the uterus, involved the open or valvular mouths of the uterine veins, and might produce purulent contamination of the system while no pus was found in the veins themselves after death. The evidence of this was in the inflammatory exudation on the inside surface of the uterus; the redness of the uterine structure, penetrating a minute distance from within outward; the symptoms of pyæmia, and the discovery of pus in distant organs. To present this idea was the chief object of his former remarks, and to give it distinctness, he had referred to and recognized the then commonly described inflammatory lesions, viz., the peritonitis, the purulent phlebitis in the uterine sinuses, and the purulent inflammation of the uterine lymphatics. These, together with endometritis, he had stated were the primary *inflammatory* lesions, and that there were other organs subject to inflammations, in a subordinate and secondary degree.*

These were some of the views that he had presented at a former meeting, and he recapitulated them now to correct any erroneous impressions which might be formed from the condensed report of his remarks published in the medical journals.

He could hardly open this subject for the further exposition of his views, without first noticing some of the statements made by Dr. Barker when the topic was last entertained by the Academy.

Dr. Barker urged that puerperal fever is a zymotic disease. So, perhaps, it may be, but Dr. Clark did not like the term, and was not in the habit of using it, because it is vague and of uncertain import. As commonly applied, it means, he said, all endemic, epidemic, and contagious diseases; and in this latitude of usage it had been made to embrace the most dissimilar affections. He had looked into the annual report of mor-

* In making this classification of the inflammations, Dr. Clark did not mean to say that they were the earliest evidence of the morbid impression made upon the system; he meant to state simply, that among the inflammatory changes, those in the peritonæum and uterus were first in time and importance.

tality in this city for the year 1842, prepared by our learned and industrious fellow-laborer, Dr. Griscom, much the best report that has ever issued from our City Inspector's office, and he finds there that the diseases usually called zymotic, are the miasmatic, typhus, and typhoid fevers,—erysipelas, small pox, scarlet fever, and measles, thrush, cholera infantum, croup, dysentery, diarrhœa, hooping cough, influenza, and syphilis. He finds that our Boston friends, in preparing the valuable mortuary reports of Massachusetts, use the term, and spread it over precisely the same ground; while puerperal fever is carried far off into another section, and appears among the diseases of the organs of generation. This he discover is the general usage of those who are attached to the word. By this very breadth of meaning, the term becomes vague and uncertain, and therefore objectionable. Should there be a disposition to use it in a stricter and narrower sense, as applicable to those diseases which reproduce the poison which causes them—in other words, strictly contagious diseases—in respect to the old idea that these diseases implied a fermentive process within the system, there would probably be less objection to the term, though even then it should be admitted into the medical language as a figure of speech, and in deference to the fathers. If the term, as used by the gentleman, means simply a contagious disease, Dr. Clark wa ready to subscribe to his opinion; but, for the sake of precision, he was compelled to prefer the words, contagious and communicable, to zymotic.

Dr. Barker had taken the occasion, he said, to speak somewhat disparagingly of the study of pathological anatomy, as if it limited our view of disease, and taught us to disregard its general history. To this Dr. Clark would reply, by pointing to what pathological anatomy had done, and the rank which pathological anatomists hold in our profession. Nobody could doubt that the immense improvements in medical diagnosis and practice, which distinguished the present century from all that had gone before it, the discovery of new diseases, and the advantageous changes in the treatment of affections long known, were to be set down to the account of this branch of study, more than to that of all others put together. New York physicians had gained the reputation of excelling their brethren in most other cities in the art of diagnosis. If they deserved this reputation, it could be attributed to nothing so much as to the general cultivation of pathological anatomy, and the reflection which that study suggests. The Pathological Society, with its profusion of means, and by its calm deliberations, and its constant reasonings from morbid appearances in the dead to symptoms in the living, was a constantly recurring lesson to teach the profession not only what this study is worth, but to inform them what phenomena in life are not illustrated by the revelations of the scalpel, and also to keep alive a love for this pursuit by a demonstration of its advantages. In this respect it is far the most useful of our public societies, and who will venture to say that it has narrowed our view of diseased action, or turned us away from the proper consideration of the morbid agencies which work unseen, but fatally, leaving few intelligible footprints?

Morbid anatomy contemplates what it cannot demonstrate to the eye, no less than the obvious changes of structure.

If we look through the catalogue of distinguished names that grace our profession in the present age, we shall find that with scarcely a single exception, they are the names of pathological anatomists, and that the foundation of their distinction is laid on this study. Brodie, and Velpeau, and Nelaton, Andral, Chomel, Louis, and Trousseau, had been nothing if their intelligence and zeal had not induced them to follow their fatal cases to the dead-house; and certainly it will not be said that these men have contracted their perceptions, and have been led to disregard any recognizable morbid agency by these pursuits. On the contrary, any just study of men must compel us to the conclusion that the same zeal which urges to the laborious use of the scalpel, is the parent equally of that industry which gives the largest and widest views of disease in all its relations.

But we are told that an appeal to the *post mortem* appearances can hardly be allowed, because the observer is likely to come with his mind preoccupied with the idea that he must find some change of structure to show for every disease. This at least seems a fair construction of the objection taken, that "pathological anatomy has led to exclusive solidism in medical doctrines." Is this really so? The present century illustrates the influence of pathological anatomy on professional opinions. Post mortem examinations were rarely obtained, or even asked for, till the time of the French revolution. Fifty years ago, the doctrines of Cullen, or rather a solidism more exclusive than his, possessed the whole medical mind. Humoralism could hardly be said to have had an advocate. Even as late as 1830, Cullen reigned. In 1835, he (Dr. Clark) was born into the profession, a solidist. Almost all the senior and middle aged men, who now complimented him with their attention, were baptized into the same faith. And where are the exclusive solidists to-day? The very phrase is obsolete, or if not, is known only to mark a period in the history of medicine. Every man's opinion has undergone a change; and pathological anatomy has effected this change. Aided by chemistry and the microscope, it has shown us, both by its positive and its negative results, that there are diseased actions which leave no marks in the solids, or only such as are secondary and subordinate. When every physician, at all familiar with post mortem examinations, has found many of the solidist opinions in which he was educated gradually melting away; when such men as Carswell, Andral, and Cruveilhier,* take the lead in investigations which show the blood to be the seat of the gravest changes, and so far as we can see primary changes, there is little ground for suspecting pathological anatomy of exclusive solidism.

But these are, after all, little more than side issues. The statement of gravest significance, and if it be supported, one which closes the argument on the real question at issue, is that the "lesions are often not sufficient to influence the progress of the disease, or to explain the cause of death." This statement, unsupported as it is by facts, is nothing more nor less than begging the question. The opinion which follows in support of this assertion, that "the most malignant form of this disease, that which proves fatal in a few hours, offers *the fewest and least striking* structural lesions," may be true enough; for intense pyæmia requires little aid to destroy life, and certainly it is not the most striking among the manifestations of morbid anatomy. Two questions are suggested by those statements:—1st. What is really the shortest time in which the most malignant form of puerperal fever destroys life; and, 2d. Whether this shortest time is not long enough for endometritis to produce fatal contamination of the blood? or, in other words, in what time purulent infection can overwhelm the vital forces?

Dr. Clark had not, as he said, made any extended researches to ascertain the shortest duration of puerperal fever. He had only looked over his own cases, and those reported by the late Dr. Vache. These latter are cases which occurred in Bellevue Hospital in 1840, when the form of of the disease was the most malignant, and the fatality among those attacked greater, than in any other visitation of the fever, since the lying-in-wards were opened. The shortest case of which he had any notes was fatal in 30 hours. The six shortest of Dr. Vache's cases lived 33, 36, 42, 44, and 48 hours respectively, and the lesions noted in these several cases were as follows:

*Case* 5.—Duration, 33 hours. All the peritoneum injected, that of the small intestines highly so; mucous lining of the small intestines much softened and inflamed; a portion of that of the stomach inflamed; a pint

* Cruveilhier, (*Anat. Path.*, Liv. xi., p. 3,) discussing this very point, viz., how purulent contamination affects the system, says: "The solidism of the school of Pinel was mute before such facts;" and adds, that the "*sympathy*" of Bichat was "a metaphor, a felicitous word, which took the place of a fact."

of straw-colored serum in peritoneal cavity; interior of uterus appeared natural.

*Case* 8.—Duration, 36 hours. Peritoneum decidedly injected, containing a pint of dirty, cream-colored, puriform fluid, with shreds of lymph; mucous membrane of stomach and intestines considerably inflamed; *the inner surface of uterus coxered with a shreddy secretion of the color of gangrenous tissue, and apparently saturated with pus.*

*Case* 19.—Duration, 42 hours. Peritoneum somewhat injected, containing a quantity of reddish-brown fluid, but no lymph; half an ounce of pus between the uterus and bladder; uterus lined with a *dark chocolate-colored stringy secretion,* which being scraped off, left *the inner surface somewhat injected.*

*Case* 12.—Duration, 44 hours. Some lymph on the peritoneum, half a pint of brown serum containing lymph flocculi, in its cavity; inner surface of uterus covered by *a fœtid stringy matter*, *intermingled here and there with a grey*, *ash-colored deposit.*

*Case* 1.—Duration, 44 hours. Peritoneum injected, a pint of sero-purulent fluid in its cavity, considerable lymph on its surface; inner surface of uterus covered by *a light red secretion, with an occasional black patch.*

*Case* 13.—Duration, 48 hours. Lymph on several portions of the peritoneum; a little pure serum in its cavity; right ovary large, and contained some small abscesses; inner surface of uterus lined with *a pink-colored matter,* of the appearance of healthy lochial discharge; broad ligaments of right side thickened and puffed out by deposition of lymph.

It is proper to state here that Dr. Clark saw all the cases reported by Dr. Vache, as they occurred after the 23d of February, up to the time the lying-in-women were sent to the island; and at Dr. Vache's request he took charge of such patients as were left in the infected wards, when new wards were opened. He also witnessed and recorded all the post-mortem examinations which were made at Bellevue after the above date. Case 4, and all from 6 to 18 he saw, and it was from his notes of the lesions that Dr. Vache's notes were partly made up. When case 5 in the above list was examined, particular attention had not been paid to the inner surface of the uterus, and the report that this surface "appeared natural," cannot be taken as conclusive, especially since Dr. Vache himself, as a rule, did not witness the dissection, for prudential reasons. It was case 8, in which the uterus presented an appearance altogether like that figured by Cruveilhier, Liv. iv., pl. vi., fig. 2, that drew attention strongly to this surface, and it was carefully studied in all the cases which occurred afterwards.

Dr. Clark holds that "the light red secretion" of case 1, the green, shreddy, gangrenous-looking effusion of case 8, the "fœtid stringy matter" of case 12, the "chocolate-colored stringy secretion" of case 19, and tho "pink-colored matter" of case 13, all indicate a morbid state of the inner surface of the uterus, and that all these "matters" and "secretions" are the fibrinous or purulent products of inflammation, colored variously by the blood in the uterus. The natural appearance of this uterine surface, for some days after delivery, is in his conviction that which is given it by adherent blood, coagulated in shreds or small masses, and by the watery secretion which is effused to wash away this blood; the prevailing color being that of dark, coagulated blood, for at least two or three days.* As to Dr. Vache's suggestion, that the "pink-colored matter," in case 13, was probably healthy lochial discharge, had he seen it himself he would hardly have entertained this idea. Dr. Clark's notes on this case are full, and, having been made at the time of the autopsy, he thinks they can be relied on. "The inner surface of the uterus was covered by a thin flocculent matter which could be easily removed. This appeared to be lymph colored by a little blood. There were a few shreds of lymph lying unattached in the neck. The odor was not very offensive." "Several vessels of the left ovary contained each, instead of blood, a drop of what appeared to be pus." Purulent contamination in this case, then, may have had a double origin. Dr. Clark adds to these, one

*Case.*—Duration, 30 hours. Peritoneum injected, most so over the uterus and intestines; about a pint of dirty or[illegible]nge-yellow, sero-purulent

---

* M. Cruveilhier (*Anat. Path.*, Liv. xiii., p. 2,) expresses the opinion that purulent effusion is in general a part of the process by which the inner surface of the uterus becomes covered again with its lining membrane He regards the lochia as a form of pus in most cases, and thinks that non-purulent lochia are the exception. He does not, however, regard the earlier lochia as having this character. It is possible that the analogy of a healing ulcer may have led him astray. With our improved means of determining this point, the lochia should be investigated anew. It has been stated in an earlier part of this discussion, that in the few cases examined of persons dying of puerperal convulsions, and other non-febrile accidents of labor, pus was not found in the uterus, and that in a portion of the cases of actual endometritis the product of the inflammatory action on the free surface is not pus, but lymph. It is possible, then, to say the least, that this distinguished observer may have erred. But if a more careful investigation should confirm his views, it would only touch one of the supposed evidences of endometritis, and not the fact of its existence or its relations to the symptoms of puerperal fever. The reparative process, though pyogenic, has no analogies in its *constitutional effects* with the inflammatory; and it appears from Sedillot's experiments that a certain moderate quantity of well-conditioned pus may really enter the circulation and yet produce no sensible effects.

matter, containing a few flocculi of lymph in the cavity; inner surface of uterus covered by a dirty pink-colored and yellowish-white exudation which appeared to be fibrine.

In all these *short* cases there was evidence of inflammatory lesion of the peritoneum; in all but one there was a morbid exudation on the inner surface of the uterus, and in this one Dr. Clark was informed by those who made the dissection that the inner surface of the organ was not particularly inspected.* There may be cases on record of shorter duration than these. But it is of little importance whether there are, or are not; for it was claimed when these views were first presented, that this internal inflammation except in the most striking cases had been overlooked. The appeal to the past, then, cannot be admitted into the argument. Future observations, made in view of the appearances here described, must decide the question, whether there is or is not a puerperal fever without some inflammatory lesion, peritoneal, uterine, or intra-uterine.

Still it will not be a loss of time, perhaps, to inquire a little farther into the probable effects of pus, laudable and ill-conditioned, mingled with the circulating blood. Sedillot, in his valuable treatise on "Purulent Infection or Pyæmia," has fixed the period in which the contamination from ordinary causes will prove fatal, at four to eight days; admitting, at the same time, the *pyoèmiées foudroyantes*, in which a large quantity of pus penetrates the circulation rapidly, as from an abscess opening into vena cava or portal vein, in which death will be determined in a few

* Cruveilhier (*Anat. Path.*, Liv. viii., pl. i. ii. iii., p. 11,) reports a case of puerperal fever (typhus puerperal) which was fatal in fifteen hours after delivery. The peritoneum contained a large quantity of white pus, such as is discharged from a phlegmonous abscess. The lymphatics of the uterus contained pus. There was dark slate-colored softening of the left lung. In this case M. Cruveilhier thinks the peritonitis preceded the delivery, as the woman had had abdominal pains and fever for five days.

On p. 10 he records a case fatal in *twenty-four hours*, the time of the attack being well marked and after delivery. The peritoneal cavity contained serum slightly bloody, and between the uterus and rectum pus. The sub-peritoneal cellular tissue was infiltrated with pus on the colon, along the right ovarian vein, in the broad ligaments, (sero-purulent matter,) on the neck of the uterus, and part of the bladder, etc. Uterine lymphatics full of pus; left ovary enlarged and softened.

In another case (p. 10) the disease was fatal in *forty-eight hours*; there was flocculent serosity in the peritoneal cavity: pus was infiltrated into the sub-peritoneal cellular tissue of the left iliac fossa; along the left ovarian vein to the kidney; around the neck of the uterus; in the walls of the vagina; and filled the lymphatics of the sides of the uterus.

In this series of cases the shortest was fatal in 24 hours. Pus was found in the uterine lymphatics in all, and in all there was peritonitis. The inner surface of the uterus does not appear to have attracted attention.

hours (p. 482.) He recognizes, also, a grave condition, indeed incurable poisoning, when pus is mingled with the blood, though the quantity be less considerable, by continual additions (p. 483); and he expressly denies that in the rapidly fatal cases, there is any formation of metastatic abscesses. In nearly all the cases he reports of this accident in man, these abscesses were found after death; but when pus was injected into the veins of dogs, *if the quantity was large*, or if he injected the *serum of ill-conditioned* pus, death occurred without such productions. His experiments also show that in the dog, at least, moderate purulent infection may produce no disastrous effects; and that in larger quantity, pus in the circulation does not always produce fatal poisoning.

These experiments are sufficiently instructive, in their bearings on several aspects of this question, to authorize a particular reference to some of them. Thus in

*Expts.* 1 and 2, M. Sedillot injected laudable pus, about 60 grains, the dogs weighing 14 and 16 lbs. troy, and it produced no sensible effect.

*Expts.* 3 and 4.—The same quantity, the dogs of the same size—for a short time yawning; pendiculations, chills, refusal of food; and in one, (a little of the pus being from a bubo), alvine evacuations; but in both prompt recovery.

*Expts.* 5 and 6.—Dog weighing 15 lbs., 22[illegible] grains of laudable pus; dog 32 lbs., 320 grains. Hurried respiration, thirst, refusal of food, disposed to be alone, lying down and unwilling or unable to rise, after some days extreme emaciation: recovery.

*Expt.* 7.—Dog 17 lbs, 120 grains of fœtid pus. Animal gravely ill; very feeble; intense chills, panting and difficult respiration. After ten days began to recover.

*Expt.* 15.—Dog 10½ lbs., 120 grains of pus, greenish and fœtid, from a phlegmon in the foot; in ten minutes animal supported himself against the wall, then sank down upon the earth, chills, hurried respiration, cries; *death in one hour.*

*Expt.* 16.—Dog 17 lbs., 60 grains of pus, from a venereal abscess of the scrotum; feebleness, difficult respiration, chills, horripulations, alvine evacuations, semi-paralysis of the posterior extremities: *death in one hour.*

*Expt.* 18.—Dog of medium size, 375 grains of well-conditioned pus; extremely feeble; stupid; when urged to walk, staggering, and falling frequently; frequent respiration; violent chills; alvine evacuations; tongue pendent; eye brilliant; later, respiration slow; then difficult, then raleuse; *death in four hours.*

*Expt.* 20.—Dog 27 lbs., 105 grains of pus from a gangrenous abscess in the muscles of the neck. Animal soon sunk to the ground; alvine evacuation; respiration accelerated, panting; attempts to vomit; neither ate nor drank; *death in five hours.*

*Expt.* 21.—An Alsacian dog, 90 grains of the same pus; anhelation; diarrhœa; refusal to eat or drink; *death in eight hours.*

*Expt.* 27.—Dog 14 lbs., 60 grains of pus from an abscess in the thigh three weeks open; violent chill; feebleness of posterior extremities; refusal of food for eight days, and of water for four; escape from the house, choosing the very cold air outside; *death in eight days.* All the internal organs healthy, but a gangrenous abscess in the right thigh.

*Expts.* 28, 29, 30, 31, 32, 33.—Successive injections of pus; all fatal.

*Expt.* 34.—Injection of 75 grains of the serum of filtered pus, repeated four times. Death on the fifth day, without metastatic abscesses; animal in good health till nineteen hours before death.

*Expt.* 37.—Dog 23 lbs., 150 grains of filtered serum from the pus of a carious bone of the leg; second and third injection; great feebleness; death on the third day, without metastatic abscess.

*Expt.* 38.—Dog 40 lbs.; injection of filtered serum of laudable pus 150 grains—no effect. The same serum on second day injected in the same quantity, no effect. Third day slightly fœtid, same quantity: animal feeble, raised himself with difficulty. Fourth day, two injections of 75 grains, now fœtid. Death eight hours after the last. No abscesses, but hard knots in the lungs

*Expt.* 41.—Successive injections of the filtered serum of pus from empyema; suppurative phlebitis; metastatic abscesses; hæmathorax; death on the eighth day.

*Expt.* 42.—Injection of 2500 grains of serum filtered pus, the pus of a lumbar abscess—no effect.

*Expt.* 44.—Dog 10 lbs. Injection of 45 grains of the globules of pus from an abscess in the arm, drawn the same day and kept at zero. Death on the third day—refusal of food; thirst; vomitings; feebleness; prolonged chills; tremblings; inflammation of the left eyelid, and of the cornea; ecchymotic spots in the lungs, but no abscesses.

The import of these experiments, and their relation to the disease we are considering, hardly require comment, especially when it is remembered that the uterine cavity is open to the ready access of air; that when inflammation has been recognized on its inner surface, it has often been of a character most likely to furnish a septic agent; and that the veins of the uterus are, after parturition, so arranged as to receive, either directly or secondarily, such septic agent, healthy or degenerated pus, in an augmenting and consequently accumulative stream.

Farther on, Dr. Clark said he might have occasion to remark on one or two of the other positions taken by Dr. Barker. It was sufficient for the present to say that the only question of importance on which they differed was—whatever might be the poisonous influence which produces the disease; foul air of an ill-ventilated or over-crowded apartment; the contagious principle given off by the sick person; the contamination of erysipelas; or, septic poison from a dead body, operating on the peculiar susceptibilities of the puerperal state; whether puerperal fever, however produced, is really ever fatal before inflammation of some sort is developed, or without the concurrent agency of such inflammation. The argument of Dr. Barker had not convinced him that the ground he had taken at an early stage of this discussion was not defensible; and he again submitted to the Academy, to be tested by their future, not by their past, experience, whether the form of puerperal fever, heretofore regarded as fatal without demonstrable lesion, is not the fever with endometritis, and consequent pyæmia or septico-pyæmia.

In turning to the treatment of puerperal fever, Dr. Clark said he was aware that he was expected to speak, not of the treatment in general, nor of the different modes of treatment adopted by physicians, but of the effects of opium. This would be the scope of his remarks, with only such additions as his observation had enabled him to make of the effects of veratrum viride and of one or two other agents. To show how his mind was led to rely on opium as a remedy in this disease, he would ask the indulgence of the Academy, while he recited the history of the exclusive opium treatment in simple and traumatic peritonitis.

The treatment of ordinary peritonitis, for which the graduates of the College of Physicians and Surgeons, and the pupils of the New York Hospital, have the sanction of those institutions, to as late a date as 1836, and probably till several years after that time, consists in bleeding, leeching, and the use of calomel and opium; the calomel, given with the view of affecting the system as promptly as possible, and the opium to retain the calomel, being given in grain and half grain doses. Indeed, these were the means relied on by nearly the whole profession. In 1841, however, visiting Woodstock, in Vermont, Dr. Clark met there Dr. B. R. Palmer and Dr. H. H. Childs, and he found that these gentlemen had become attached to Armstrong's method, as it is commonly called. He saw, with Dr. Palmer, patients treated in that way doing much better than on the more generally adopted plan. In the spring of 1841–'2 or '3, probably in one of the earlier years, talking of these cases, Dr. Clark suggested to Dr. Palmer that probably opium was the curative agent in peritonitis, and that the bleeding might be safely omitted, if the effects of the drug were steadily kept up. The next season, when they met, Dr. Clark was able to report an increased confidence in his suggestion, for he had tried opium alone, and had been successful. This statement he makes on the authority of Dr. Palmer, for in the lapse of years he had himself quite forgotten how the thought originated. From that time, neither he nor Dr. Palmer had bled a patient with this disease, and he remembers but one instance in his own practice in which leeches have been used; and Dr. Palmer is now in the habit of saying, that with this treatment, peritonitis is no more formidable than a pneumonia. Up to 1850, Dr. Clark had treated eight cases successfully. In that year he saw his first unsuccessful case. The subject of the disease was a gentleman of some distinction, seen with Drs. Bulkley and J. M. Smith. In that case, however, it is proper to add, the disease probably gained much force, under the mask of diarrhœa, before its true nature was recognized. Since that time he has lost but two in private practice, one in the practice of Docter Gilman, and one in that of Dr. McNulty. In the first of these, there were some grounds for suspecting perforation, and in the second that accident was demonstrated after death. In the hospital there has, perhaps, been one death from the same cause in that period.

It was not till he had tried the opium in puerperal peritonitis that he became aware that his experience was but a confirmation of earlier observations; but it soothes his vanity somewhat, that among the many physicians here and elsewhere who talked about and adopted the treatment, there were none who, at that time, could enlighten his ignorance. The report of what Drs. Graves and Stokes had done, in the fifth volume of the Dublin Hospital Reports, had either not been seen by New York physicians, or had attracted but little attention, and had been forgotten. It is certain that the suggestions of Drs. Graves and Stokes were not adopted by the profession here, and, so far as is known, had not influenced the opinions of any practitioner in the city. In Dr. Grave's "Clinical Lectures," by Neligan (p. 244, vol. ii.) the following passage occurs: "The first case in which I used opium in peritonitis occurred in 1822, in the old Meath Hospital. It was that of a woman in whom the inflammation set in after the operation of tapping for dropsy. The case seemed so hopeless, and the suffering of the patient so intense that I was induced to order opium for her in large doses. She also got wine. To my astonishment, she recovered. I afterwards published with Dr. Stokes our conjoined experience of the efficacy of this plan of treatment, in the fifth volume of the Dublin Hospital Reports." "The use of opium in the form of peritonitis then described, is almost universally adopted," (that is, almost universally in Ireland.) Dr. Graves, then, did not propose opium as a remedy for *peritonitis*, but for peritonitis *of a particular form.*

The following extracts from Armstrong's Lectures will explain his plan of using opium: 1. "The first and main remedy is blood-letting, carried to approaching syncope." 2. "As long as the tongue continues moist, opium with blood-letting may be considered a sovereign remedy." "As soon as the patient recovers srom the syncope, give him, if an adult, three to five grains of opium." He goes on to say that if in four hours the pain returns, the bleeding should be repeated, and the patient should take two grains of opium, with calomel; and if two hours later there is still pain, he directs still another bleeding, and one and a half or two grains of opium, with calomel. "The opium," he says, "tends to arrest the secretions of the liver, but when combined with calomel, it has not that effect. Whenever, therefore, you repeat the opium, give calomel with it." The estimation in which Dr. Armstrong holds opium is emphatically announced in the following citation: "If I were subject, etc., and were only allowed to have opium or blood-letting, I would choose opium, though I would prefer both together."

Thus it appears that Graves proposed the use of opium for a particular form of peritonitis only, and Armstrong used it only as an auxiliary, though regarding it as rather the more benevolent agent among those he had chosen. It is not improper to add that the latter author startles our confidence a little, by a somewhat braggart air, and by figures that appear to us very large. He says: "I have treated nearly three hundred cases, with a success far greater than I have had from any other plan; and I could defy all the physicians of this country to show any more successtul practice." Dr. Watson, in his Lectures (Am. Ed., 1847, p. 737), referring to the use of opium in peritonitis, says that—"A pamphlet was published some years ago, by Mr. Bates, of Sudbury, recording some striking cases of recovery from severe peritonitis, under large and frequent doses of opium, and a rigid adherence to the horizontal position." It is not stated whether bleeding was resorted to in those cases or not. But Dr. Watson is not convinced of the propriety of relying on opium, either by Mr. Bates's pamphlet, or by the cases of Drs. Graves and Stokes, which he reviews; for he says: "To sum up, then, bleeding, and calomel, and opium are to be resorted to for checking the inflammation." "The opium," he says, "allays pain, and perhaps relaxes spasm; mercury tends to arrest the inflammatory action." Dr. Clark presented the facts in this form, to secure for the opium treatment all the importance to which such independent and concurring observations should fairly entitle it.

But the history is not yet concluded, for said Dr. Clark, if I may not claim for myself the priority of the discovery, by just so much as I esteem it useful, I am happy to be able still to claim it for my country. To you, sir, [addressing the president], our acknowledgments are due for having preserved on record the fact that the late Dr. Wright Post, of this city used this *magnum donum Dei*, to combat inflammations, before either of the physicians, whose authority is now so often quoted. Dr. Post, it seems, used opium in large doses "for its paralyzing influence over disease" of an inflammatory character, as early as 1804, and for enteritis in 1810, perhaps considerably earlier; for he seems to have been familiar with its use at that date. This pamphlet was published in 1829, before I was myself a student of medicine, and I became indebted to Dr. Van Buren, two or three years ago, for my first opportunity of perusing it. It is entitled a "Biographical Memoir of Wright Post, M.D., late Professor of Anatomy, etc., by Valentine Mott, M. D.," etc.; and contains a letter from Dr. F. G. King, addressed to the author, which details these facts. It is worthy of republication at this time, when the virtues of opium are undergoing renewed investigation.*

The details of this opium treatment of peritonitis are so nearly identical with those of the same treatment applied to puerperal fever, that the management of the two diseases can be most conveniently spoken of together; and the hour being far advanced, it is better that both be postponed till another occasion. It is, perhaps, proper to say in closing, and in anticipation of what is to be said hereafter, that my confidence in the opium treatment of puerperal fever, with peritoneal complication, is in no degree shaken by accumulating experience, but is rather increased; while its usefulness in that form of the disease which is attended by purulent infection, has not been demonstrated, at least, as an exclusive method.

New York, Feb. 5th, 1829.

My dear Sir:—In reply to your inquiries as to the use of anodynes and opium by the late Dr. Post, I have to remark, that in conversation with him some two years past, relative to Dr. Armstrong's practice in inflammatory diseases, he told me that the use of opium, as recommended by that gentleman, (except in *larger doses*,) was corroborated by his own experience for a long series of years, and that to him it was by no means a novelty: for that in 1804, he was called to a child about three years of age, suffering under a violent pneumonic attack, accompanied by pain, cough, and febrile excitement. That he accordingly bled, blistered and evacuated the patient, afterwards placing him under the use of antimo-

*See Dr. King's letter to Dr. Mott.

nials, but all without benefit. Matters proceeded from bad to worse, until the child, exhausted by constant cough and excessive restlessness, seemed nearly at the point of death. Under these circumstances, he determined to quiet all these irritating symptoms by a powerful anodyne, and accordingly exhibited 60 drops of laudanum. Two hours after, he was called to the child, then supposed by its parents to be dying. He found the features sunken, the surface covered with a cold clammy sweat, and secretions of an unpleasant appearance about the eyes and nostrils, but the pulse had diminished in frequency, and was more full; the respiration was slower, and everything indicated the full and desired action of the anodyne. The parents were astonished to hear the physician say that the child would soon be better. The next morning all untoward symptoms had subsided, and the child became rapidly convalescent and recovered.

This was his first trial of anodynes in such affections; his *experiment*, if you please; but a few months afterwards, a similar case occurring, he he immediately resorted to the anodyne; depletion and evacuants having been premised, and with similar success, since which period he has generally continued that mode of practice; latterly, however, substituting the Dover's powder in place of laudanum, in pneumonic attacks.

In 1810, he was called in consultation upon a gentleman in Jersey, suffering under enteritis. He found that he had been repeatedly bled, blistered, and evacuated, but to no advantage; the pain still continued acute; the pulse was small, frequent and corded; the skin dry and hot. Under these circumstances he suggested the propriety of exhibiting a powerful anodyne, in order to quiet all irritation and give nature an opportunity of recovering herself. After a little hesitation on the part of the attending physician, it was finally determined to adopt the course proposed, and 100 drops of laudanum were directed; an hour elapsed,—no sensible effect having been produced, when the *dose was repeated*, and in half an hour the patient was under its full influence. He awoke the next day free from pain or tenderness, and so recovered. The same gentleman has been frequently attacked since with the same affection, and uniformly after being bled and evacuated, he has recourse to his anodyne, which rarely fails to quell the disease. But, to be efficacious, the dose must be *heroic*, at least such was the opinion of Dr. Post, who often remarked that practitioners, especially in England and France, were not aware of the value of opium in inflammatory diseases, for even when employing it in such cases, their doses were too trivial to exert any marked influence over the malady. He himself always exhibited it under the opinion, that to obtain its soothing effect upon the system, and its *para-*

*lyzing* influence over the disease, it must be given in large doses. In diarrhœa and certain conditions of dysentery, after having cleansed the passages, he employed laudanum or Dover's powder with the happiest effect; in fact, he rarely used much else than salts and Dover's powder in diarrhœa, in adults. In his own case, he was no less prodigal of anodynes than with his patients. Being, as you well know, for many years a constant prey to pleuritic affections, his troatment of himself was short and efficacious, viz., blisters and purgatives, followed by 80 or 100 drops of laudanum, which quieted his cough—allayed pain, and soon placed him in condition to resume his business.

In conclusion, permit me to state an oocurrence which took place under my own eyes, two years previous to his death. He was then violently attacked with pleurisy, accompanied with much fever, for which he had been purged and blistered, and at the period in question, was under the use of antimonials. At this time he directed me to give him 70 drops of laudanum. I remonstrated, directing his attention to the dryness of his skin, its increased heat, and the frequency and hardness of his pulse. His answer was, "believe in my experience rather than in your theory; give me 70 or 80 drops of laudanum, and an hour will convince you of its propriety." It was given, and within the hour his pulse became calm, full and slow; his skin was covered with a gentle perspiration, and his condition strikingly improved. He left his bed the next day, and frequently since has said to me, "I think I have given you a clinical lecture that you will remember."

Such, my dear sir, are the facts concerning which we some time since conversed, and if they can be of any service to you, employ them as you think proper.

Yours truly,

F. G. KING.

To. V. Mott, M. D.

